# Dental management of patients with cancer across the care pathway: A scoping review of clinical protocols

**DOI:** 10.4317/medoral.27886

**Published:** 2026-01-24

**Authors:** Fernando Mauricio Espada-Salgado, María Mihaela Iuga, Alex Rodrigo A Capellino-Gambetta

**Affiliations:** 1 Faculty of Health Sciences, Private University of Tacna, Tacna, Peru; 2 Faculty of Health Sciences, Jorge Basadre Grohmann National University, Tacna, Peru

## Abstract

**Background:**

Oral complications of cancer and its therapies impair quality of life and can disrupt oncologic care. Standardizing dental management across the care pathway is essential. Objective: To map operational protocols, guidelines, algorithms, or structured recommendations for dental management of oncology patients across pre-treatment, active treatment, and follow-up or survivorship from 2000 to 2025.

**Material and Methods:**

Scoping review according to JBI guidance and reported with PRISMA-ScR. Searches were run in PubMed or MEDLINE, SciELO, Scopus, Web of Science, and the Virtual Health Library on October 19, 2025, without language restrictions. Dual screening was performed in Rayyan. Data were charted in a standardized per-record matrix and synthesized narratively, in tables, and with graphics by care phase and oncologic modality. No formal critical appraisal or meta-analysis was undertaken.

**Results:**

Twelve records were included. Coverage concentrated on pre-treatment (11 of 12; 91.7 percent), followed by follow-up or survivorship (6 of 12; 50.0 percent) and active treatment (3 of 12; 25.0 percent). Convergent recommendations included comprehensive pre-treatment assessment with sanitation, an intensified prevention bundle with hygiene instruction and topical fluorides, tooth-prognosis-based extraction criteria with defined healing windows, and supportive measures during therapy for mucositis, xerostomia, and infections. Gaps included limited standardization of survivorship pathways, insufficient personalization by oncologic modality (radiotherapy, chemotherapy, HSCT or CAR-T, immunotherapies), and scarce operational detail on safe timing windows, clinical or hematologic cutoffs, and referral or consultation triggers.

**Conclusions:**

Evidence supports an operational consensus for pre-treatment clearance and a core supportive bundle during therapy. To optimize safety and outcomes, particularly in middle-income settings, modality-specific protocols with explicit dentistry-oncology referral pathways, dose- and hematology-informed decision rules, and defined follow-up schedules are warranted.

## Introduction

Cancer patients frequently develop oral complications arising both from the disease and from its treatment. Conventional cytotoxic chemotherapy, radiotherapy, targeted agents, and immunotherapies can precipitate oral mucositis, xerostomia, opportunistic infections, dental caries, taste dysfunction, and osteoradionecrosis, collectively compromising oral function, nutrition, and quality of life. Oral mucositis is among the most prevalent toxicities of cancer therapy, affecting about 40% of patients receiving standard chemotherapy, increasing to approximately 76% with high-dose chemotherapy before hematopoietic stem cell transplantation, ranging from 30% to 60% among patients undergoing head and neck radiotherapy, and approaching 90% with concurrent chemoradiation (1 , 2).

Baseline dental disease is common before oncologic treatment, particularly in head and neck cancer. In a clinical registry, more than one third of patients had untreated caries, nearly half presented with periodontal pockets 5 mm or deeper, and approximately 50% required dental treatment most often extractions before radiotherapy (3). Beyond mucositis, long-term sequelae of head and neck radiotherapy, especially salivary gland hypofunction and xerostomia, further impair oral health and quality of life (4). Severe mucositis is clinically important because pain, weight loss, and impaired oral intake often require urgent care or hospitalization and may lead to dose reductions, unplanned interruptions of radiotherapy, or discontinuation of chemotherapy, potentially jeopardizing cancer control and increasing costs; accordingly, scientific societies advocate structured oral care protocols across the cancer pathway, and MASCC/ISOO guidelines recommend meticulous oral hygiene, fluoride use, tooth brushing and interdental cleaning, alcohol-free rinses, patient education, and follow-up to prevent or mitigate mucositis during cancer therapy (5).

Although targeted therapies and immune checkpoint inhibitors offer greater tumor selectivity, they still produce distinctive oral toxicities, such as mucosal lesions, salivary dysfunction, and neuropathic pain, that increase morbidity and require tailored dental strategies (6). Consistent with the high burden of pre-treatment needs, a retrospective cohort of mixed tumor types reported substantial dental treatment requirements before therapy initiation, including frequent indications for extractions to reduce infection risk and optimize oral function (7).

Mechanistic and clinical syntheses of treatment-related mucosal injury emphasize heterogeneity across anticancer regimens and patient contexts, which contributes to variability in recommendations and implementation (8 , 9). Published protocols also differ in content, timing, and operational detail across settings, often placing stronger emphasis on pre-treatment interventions than on measures during active therapy or structured survivorship follow-up (10).

This scoping review therefore aims to map and synthesize clinical protocols, guidelines, algorithms, and structured recommendations for dental management at pre-treatment, active-treatment, and follow-up stages from 2000 to 2025, identifying areas of convergence, operational gaps, and heterogeneity across oncologic modalities to inform clinical practice and future research.

## Material and Methods

Methodological approach

This scoping review followed Joanna Briggs Institute (JBI) methodology, appropriate for mapping broad and heterogeneous evidence and for identifying gaps to inform practice and research (11). Reporting adhered to the PRISMA-ScR checklist and explanation (12).

Consistent with JBI guidance for scoping reviews, no formal risk-of-bias appraisal was undertaken and findings are presented descriptively.

Protocol and registration

The protocol was prospectively registered on the Open Science Framework (OSF) on 20 October 2025. The registration record, full search strategies, screening logs, data-charting templates and analytic outputs are openly available (OSF registration DOI: 10.17605/OSF.IO/6RHYM). Any deviations from the protocol are documented in the OSF record and do not alter the review objectives.

Review question and PCC framework

Population: Individuals with a diagnosis of cancer of any age.

Concept: Clinical protocols, clinical practice guidelines, algorithms or structured and actionable recommendations for dental management.

Context: Dental care across pre-treatment, active treatment and follow-up or survivorship.

Review question: Which clinical protocols have been published from January 2000 to October 2025 for the dental management of oncology patients across pre-treatment, active treatment and follow-up phases?

Eligibility criteria

Inclusion: Peer-reviewed documents that explicitly presented structured and actionable clinical protocols, practice guidelines, expert consensus statements or primary studies with an operational algorithm for the dental management of oncology patients (pediatric or adult; solid tumors or hematologic malignancies), applicable to any care phase and clinical setting. No language restrictions were applied.

Exclusion: Single case reports; letters, editorials or narrative reviews that did not provide a clearly structured protocol or algorithm; conference abstracts or posters without a peer-reviewed full text; studies focused only on manifestations, prevalence or pathophysiology of oral conditions in oncology without explicit, actionable management guidance; and documents on non-oncologic populations unless they included specific dental recommendations formulated for cancer patients. Records for which the full text could not be obtained were also excluded. In cases of duplicate or overlapping publications, the most complete and recent version was retained for data charting.

Information sources and search strategy

Five databases were searched systematically: PubMed/MEDLINE, SciELO, Scopus, Web of Science and the Virtual Health Library (BVS, Complete Collection). Searches combined controlled vocabulary (MeSH, Entree, DeCS) and free-text terms tailored to each source. Example PubMed syntax: ("oral care" OR "dental management" OR "oral health") AND (cancer OR oncology OR neoplasm*) AND (guideline OR protocol OR "clinical pathway" OR algorithm).

Searches were last run on 19 October 2025. Reference lists of included articles were screened for additional eligible peer-reviewed documents. Full search strings and any refinements are available in OSF.

Selection of sources of evidence

Records were managed in Zotero for de-duplication. Two reviewers independently screened titles and abstracts, followed by full texts, using Rayyan (13). Discrepancies were resolved by consensus or a third reviewer. Reasons for full-text exclusion were recorded. The selection process is summarized in the PRISMA-ScR flow diagram.

Data charting

A piloted form captured: Author, year, country; document type; oncology target (solid or hematologic; adult or pediatric); care phases addressed; recommended dental interventions (for example, prevention and topical fluorides; dental clearance and timing for extractions; management of mucositis, xerostomia and infections; prosthetic rehabilitation; follow-up frequency) and clinical or operational rationale. One reviewer charted data and a second verified entries; disagreements were resolved by consensus or arbitration.

A per-record data-charting matrix captured citation, document type, issuing body/country and year, oncologic modality (surgery, radiotherapy, chemotherapy, targeted/immunotherapy, HSCT/CAR-T), phase(s) of care (pre-treatment, active treatment, follow-up/survivorship), setting, and key protocol components (assessment/clearance, prevention, extraction criteria and healing windows, supportive measures, operational thresholds, and referral triggers). This matrix is available as Supplementary Table S1 (http://www.medicina.oral.com/carpeta/suppl1_27886).

Synthesis of results

Findings were synthesized narratively and in tables, grouped by care phase and oncologic modality. When available, frequencies of interventions were summarized and simple graphics prepared (bar chart by phase; heatmap for intervention by modality) to aid comparison. In accordance with JBI guidance for scoping reviews, no formal critical appraisal or meta-analysis was undertaken; reported outcomes or indicators were summarized descriptively (11).

Data availability

All materials supporting this review, including full search strategies, screening logs, the PRISMA-ScR checklist, data-charting templates and analytic outputs, are available in OSF (registration DOI: 10.17605/OSF.IO/6RHYM).

## Results

Selection and general characteristics of the documents

**Figure 1:**
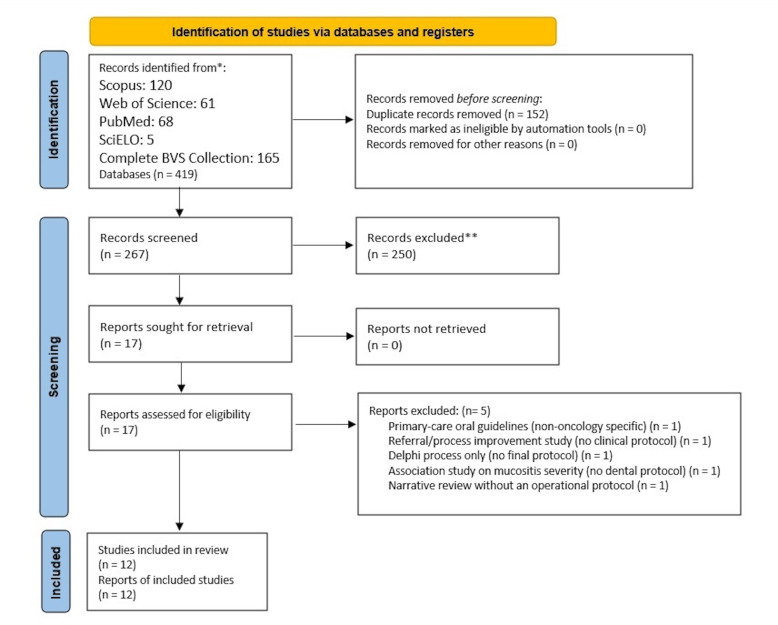
PRISMA-ScR flow diagram for the scoping review. Searches conducted on 19 October 2025.

We screened 419 records and included 12 documents (Figure 1). Documents addressing dental management in the context of head and neck radiotherapy included guidelines or consensus statements, institutional protocols, and observational cohorts (14 - 18). Documents focused on dental evaluation and management in hematologic settings were also included, covering pre-HSCT care, pediatric oncology, and hematologic malignancies including CAR T-cell therapy (19 - 21). Additional documents addressed dental management before head and neck radiotherapy and related supportive care considerations, with particular emphasis on prevention of complications such as osteoradionecrosis (22 - 25).

1

Detailed characteristics for each included record are summarized in Supplementary Table S1 (http://www.medicina.oral.com/carpeta/suppl1_27886).

Coverage by phase of care

**Figure 2:**
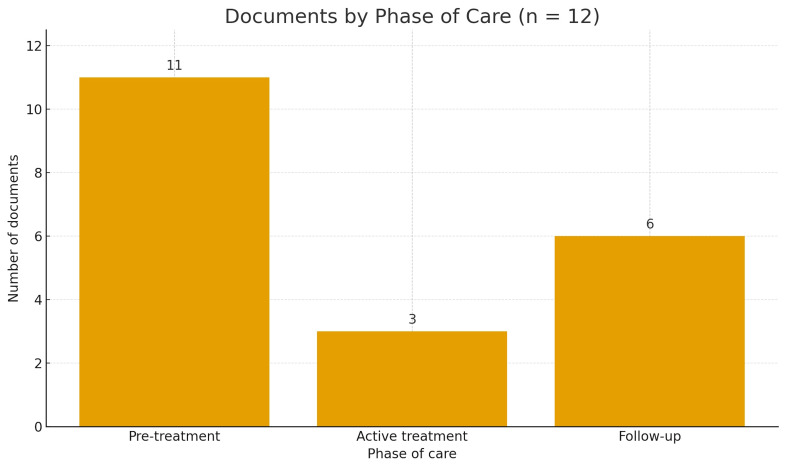
Distribution of included documents by phase of care (pre-treatment, active treatment, follow-up; n=12).

Coverage focused on pre-treatment (11 of 12; 91.7%), followed by follow-up/survivorship (6 of 12; 50.0%) and active treatment (3 of 12; 25.0%). This distribution is shown in Figure 2 and guides the phase-based synthesis below. See Table 1 for the intervention-by-phase matrix.

2

Table 1

Phase-based synthesis

1. Pre-treatment

Head and neck radiotherapy sources emphasize standardized pre-treatment care, including comprehensive dental evaluation (history; intraoral and extraoral examination; periodontal and caries status; third molars; and imaging when indicated, such as OPG or CBCT) and structured preventive education (14 - 17). Hematologic oncology and HSCT sources, including the MASCC/ISOO clinical practice statement, prioritize pre-treatment dental clearance before cytoreductive chemotherapy or CAR T-cell therapy to reduce infectious complications and minimize treatment interruptions (19 , 21). Pediatric guidance similarly recommends completing necessary dental care before immunosuppression or radiotherapy whenever feasible; otherwise, it prioritizes infection control, indicated extractions, and preventive measures such as fluoride, dietary counseling, and oral hygiene support (20).

In addition, several head and neck radiotherapy documents provide sanitation strategies with tooth-level decision rules and dose-informed extraction thresholds, prioritizing high-risk teeth such as mandibular molars with advanced periodontal disease and symptomatic third molars when adequate healing time is available (22 , 23). When extractions are required, a healing interval of about 14 days before radiotherapy initiation is commonly recommended (24).

2. Active treatment (RT, CT, HSCT, CAR-T)

During active cancer treatment, protocols converge on intensified oral hygiene and close monitoring. In head and neck radiotherapy, recommended measures include daily topical fluorides, preferably delivered with custom trays, alcohol-free rinses, and targeted management of mucositis and xerostomia, with regular clinical reviews during treatment (14 , 17). Evidence from an oropharyngeal squamous cell carcinoma cohort highlights the need for risk-adapted monitoring, as the five-year cumulative incidence of post-radiotherapy extractions reached 30.7 percent, largely driven by radiation caries and periodontal disease, with higher risk observed among smokers, patients with poor oral hygiene, and those receiving higher mandibular and parotid doses (18).

In hematologic oncology settings, including HSCT and CAR T-cell therapy, invasive dental procedures are generally avoided during periods of neutropenia or thrombocytopenia. Management focuses on pain control, treatment of opportunistic infections, and guideline-based care for oral mucositis, in alignment with established consensus recommendations and MASCC/ISOO guidance (19 , 21).

3. Post-treatment and follow-up

After head and neck radiotherapy, follow-up care prioritizes prevention and cautious procedural planning; extractions are generally avoided during the first year unless unavoidable, and long-term fluoride-tray use is maintained to reduce caries risk (24). Osteoradionecrosis incidence may be low when standardized dental pathways are integrated with modern radiotherapy, with one single-center series reporting 2.3 percent (17). After HSCT or CAR T-cell therapy, elective dental care can resume once hematologic and immune recovery is achieved, with continued surveillance and management of mucositis, candidiasis, hyposalivation, and oral graft-versus-host disease in coordination with oncology (19 , 21).

Modality-based synthesis

Head and neck radiotherapy. Across documents, tooth-level decisions are guided by the planned radiation dose together with periodontal and caries status, overall prognosis, and anticipated maintainability. There is a consistent tendency to extract high-risk teeth, particularly mandibular molars with advanced periodontitis and symptomatic third molars, when adequate healing time is available (14 , 17 , 22 , 23).

**Figure 3:**
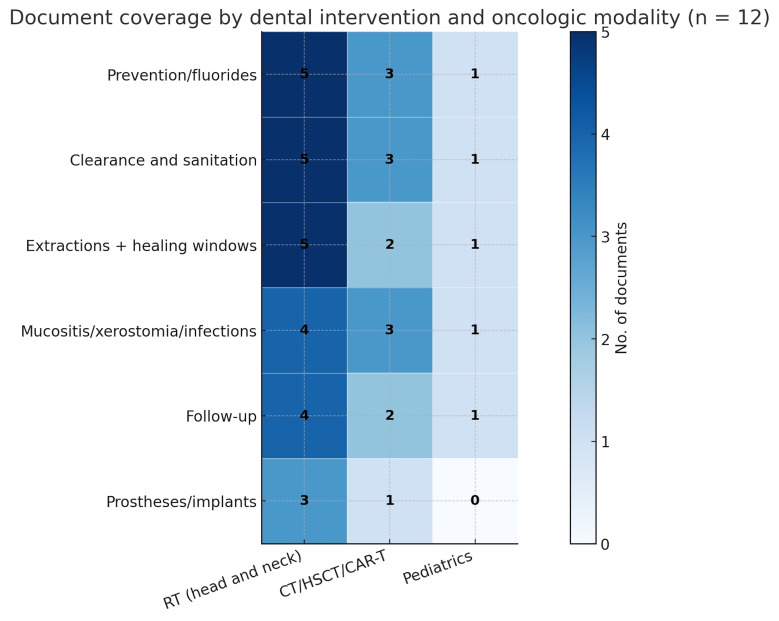
Intervention coverage by modality (n=12). Cells show document counts. Modalities: H&amp;N RT, CT, HSCT/CAR-T, Pediatrics.

Chemotherapy, HSCT, and CAR T-cell therapy. In hematologic settings, pre-treatment evaluation and oral sanitation aim to reduce infectious complications and acute dental emergencies during therapy. The MASCC/ISOO clinical practice statement outlines prioritization and timing of dental procedures before and after treatment. Figure 3 summarizes the intervention-by-modality matrix across the 12 included documents (19 , 21).

3

Pediatrics. Pediatric guidance follows the same core principles, adapted to dental development. Whenever feasible, necessary dental care should be completed before immunosuppression or radiotherapy; otherwise, management prioritizes infection control and prevention, including fluoride, oral hygiene support, and dietary counseling (20).

Recurrent interventions and operational features

Prevention and supportive care. Across documents, preventive education and supportive measures are consistently emphasized, including oral hygiene and dietary counseling, fluoride use with custom trays during radiotherapy, trismus physiotherapy, and targeted management of mucositis, xerostomia, and oral infections (17 , 24).

Extraction criteria. Indications for extraction are based on tooth-level risk assessment that integrates estimated radiation dose, non-restorable caries, advanced mobility or severe periodontitis, and symptomatic third molars, with careful consideration of adequate healing intervals before radiotherapy (14 , 17 , 22 - 24).

Reported outcomes. When standardized dental protocols are combined with modern radiotherapy planning, reported osteoradionecrosis rates are low, with one single-center series documenting an incidence of 2.3 percent (17). Longer-term observational data indicate that post-radiotherapy extractions remain frequent, with a five-year cumulative incidence of 30.7 percent associated with smoking, alcohol use, poor oral hygiene, and higher mandibular or parotid doses (18). In socioeconomically vulnerable populations, extensive pre-radiotherapy extractions did not fully prevent osteoradionecrosis, supporting a multifactorial etiology (22). Beyond outcomes, several protocols explicitly describe therapeutic approaches for oral mucositis and osteoradionecrosis; Table 2 summarizes these regimens, highlighting key agents, reported responses, and protocol-level differences across the 12 included documents.

Table 2

## Discussion

This scoping review shows that published dental protocols in oncology broadly align with international supportive-care guidance, while highlighting persistent operational gaps across the cancer care pathway. Pre-treatment recommendations converge on comprehensive dental assessment and dental clearance with early referral to allow adequate healing before oncologic therapy, particularly in head and neck radiotherapy (14 - 24). This approach is reinforced by a dedicated clinical practice guideline and a recent review emphasizing elimination of oral infection foci and coordination with oncology timelines (25 , 26). In hematologic malignancies and CAR T-cell therapy, the MASCC/ISOO clinical practice statement similarly stresses timely pre-treatment dental evaluation to permit mucosal healing and alignment with systemic treatment plans (21).

During active therapy, prevention and supportive care predominate. Basic oral care, including professional cleaning, toothbrushing, interdental cleaning, alcohol-free rinses, and topical fluorides, is consistently recommended and supported by systematic-review evidence across chemotherapy, radiotherapy, and HSCT settings (27 - 30). Adjuncts with evidence include oral cryotherapy, photobiomodulation, and benzydamine mouthwash, which should complement, not replace, consistent basic oral care delivered with close follow-up and effective oncology-dentistry communication (5 , 27 , 28).

Three gaps remain evident. Survivorship follow-up is poorly standardized, with few protocols specifying visit frequency, content, or risk markers despite ongoing risks of radiation caries, periodontal breakdown, and osteoradionecrosis in head and neck cancer survivors (16 - 18 , 24 , 26). Modality-specific personalization is limited, and actionable operational detail is often insufficient, with few sources specifying explicit timing rules, hematologic thresholds, or referral triggers, despite acknowledging healing intervals and cytopenia-related constraints (5 , 14 , 21 , 24).

Methodological strengths include adherence to JBI and PRISMA-ScR guidance, dual independent screening, and synthesis structured by phase of care and modality; limitations inherent to scoping reviews apply, including the absence of risk-of-bias assessment and the inability to pool heterogeneous evidence (11 , 12). Implementation in Latin America and other middle-income settings requires contextualization, but global policy attention to oral health within NCD and universal health coverage agendas supports opportunities to embed feasible dental pathways, prioritize cost-effective measures, and formalize referral routes supported by structured follow-up (29).

## Conclusions

An operational consensus emerges from the literature: Comprehensive pre-treatment dental evaluation, focal sanitation and intensive education are regarded as safety standards before CT, RT, HSCT or CAR-T. During therapy, a core bundle of enhanced hygiene, non-alcoholic rinses, topical fluorides, oral cryotherapy, photobiomodulation and benzydamine (in appropriate head-and-neck RT settings) helps mitigate oral toxicity when delivered within structured oral-care protocols.

Key gaps persist. Survivorship follow-up needs clearer standardization (visit frequency, clinical content and risk markers), recommendations should be further tailored to oncologic modality and individual risk, and routine practice requires better operational detail, including explicit timing windows, clinical and hematologic thresholds and bidirectional referral criteria to support coordinated dentistry-oncology care across the entire pathway.

## Figures and Tables

**Table 1:** Table Main dental interventions and their phase-of-care recommendation.

Intervention / Phase	Pre-treatment	Active treatment	Follow-up
Clinical-radiographic assessment & risk	Yes	-	Yes
Hygiene education; topical fluorides	Yes	Yes	Yes
Sanitation (short restorative/periodontal)	Yes	-	Yes
Extraction of poor-prognosis teeth & waiting times	Yes	-	(only if essential after recovery)
Management of mucositis / xerostomia / infections	-	Yes	Yes
Prostheses / implants	-	-	Delay and assess risk (RT)

a) Extractions in follow-up only if indispensable, after resolution of infection and tissue/hematologic recovery, considering delivered dose and individual risk. b) Prostheses/implants in irradiated patients: Consider postponement (e.g., removable prostheses ≈6-12 months; implants ≥12 months) according to clinical evaluation and osteoradionecrosis risk.

**Table 2:** Table Treatment modalities for oral mucositis and osteoradionecrosis across included protocols.

Author/Year/Type	Condition	Treatment modalities & agents	Reported response	Protocol differences
Caribé-Gomes et al. 2003 (review)	Mucositis & ORN	OM: Saline/bicarbonate ± H₂O₂/CHX; coating agents; topical anesthetics; systemic analgesics/antibiotics. ORN: Avoid trauma; irrigation + antibiotics; tetracycline/CHX; HBO or resection.	Symptom relief and infection control (no outcome data).	Older narrative; several therapies now outdated.
Bonan et al. 2006 (pre-RT cohort)	ORN	Extensive pre-RT extractions; daily 1.23% NaF gel; hygiene advice; no dentures during RT; ORN managed with irrigation, sequestrectomy ± HBO.	ORN 21%; radiation caries 11%; partial healing with conservative care/HBO.	Low-income HNC cohort; many extractions close to RT start; multifactorial ORN; older protocol.
Bonar Álvarez et al. 2018 (pre-RT cohort)	ORN prevention	Full exam; scaling/restorations; extract poor-prognosis teeth; 0.12% CHX; antibiotics; daily fluoride; saliva/Candida tests; hygiene instruction.	Most patients required extractions (mean 3.7) and scaling; no toxicity outcomes reported.	Recommends 2-6 wk healing; no specific mucositis drugs; focus on caries and perio control.
White et al. 2019 (VA consensus)	Mucositis; ORN	Pre-RT: Extraction algorithm, 10-21 d healing, daily high-NaF; saliva substitutes ± pilocarpine. OM: Saline/bicarbonate, honey/ice, KGF/G-CSF, IV glutamine, laser, sucralfate, PTA. ORN: Avoid post-RT surgery/dentures; HBO if surgery needed.	Goal: Reduce ORN, radiation caries, OM and xerostomia (no direct outcome data).	US VA system; evidence + Cochrane-based; structured workflow and lifelong maintenance.
Piret & Coucke 2021 (protocol)	Mucositis & ORN	Pre-RT: Exam, selective extractions, smoothing/suture, ≥3-wk healing, high-F trays. During RT: Soft brush, anaesthetic + HC rinses, no extractions. Post-RT: Daily 12,500-ppm F trays, strict hygiene, endo + antibiotics, extractions last resort.	Aim: Limit caries, OM pain and ORN; preserve function and QoL.	HNC RT population; integrates IMRT/VMAT dose and surgery timing; stresses long-term follow-up.
Watson et al. 2021 (CDON pre-RT guideline)	ORN prevention; mucositis	Dose-based extractions (70 Gy maxilla; 60 Gy mandible); 7-14 d healing; full dental exam; diet advice; daily fluoride; 3-monthly cleanings; bland rinses; avoid irritants.	Intended reduction of post-RT ORN, tooth loss and caries (no direct data).	Delphi national guideline; strong ORN focus; limited mucositis pharmacotherapy.
Bhandari et al. 2022 (pre-RT protocol)	ORN prevention; mucositis	3-4 wk pre-RT workflow; multi-quadrant extractions + alveolectomy with sutures/antibiotics; ≥2-wk healing; perio care/restorations; daily 1% NaF gel or 0.05% rinse; fluoride varnish q3 months; exercises; 3-monthly follow-up; cautious denture use.	Designed to limit ORN, caries and trismus and keep RT on schedule (no outcome data).	Low-resource HNC setting; strong focus on timing and logistics; little mucositis pharmacology.
Hoffmann et al. 2022(pre-RT cohort)	ORN prevention	Standardised pre-RT screening; extract poor-prognosis teeth; perio therapy/restorations; smoothing sharp edges; professional cleaning; fluoride trays/splints.	Extractions in 56% (mean 4.4 teeth); ORN 2.3%; stomatitis 37%; xerostomia 29%.	IMRT/VMAT; low ORN with intensive pre-RT care; no OM drugs.
Correa et al. 2023 (Brazil pre-HSCT consensus)	Mucositis	Reinforced hygiene; CHX rinse; topical fluoride; removal of orthodontic appliances; timed perio care/extractions ± antibiotics.	Aims to reduce early HCT oral infections and trauma (no direct outcome data).	Pre-HSCT only; MASCC/ISOO-aligned consensus; RT-related ORN not addressed.
AAPD 2024 (guideline)	Mucositis	Hygiene; saline/bicarbonate rinses; benzydamine; oral cryotherapy; palifermin; PBM; coating agents; topical anaesthetics.	Lower incidence, severity and pain of OM.	Paediatric focus; extrapolated from adult trials; variable strength of evidence.
Chin et al. 2024 (OPSCC cohort)	Post-RT extractions & ORN risk	Risk-based pre-RT screening; weekly fluoride trays during RT; intensive follow-up; post-RT extractions >40 Gy with prolonged antibiotics ± HBO.	Post-RT extraction 30.7%; 26 ORN cases (mostly mandible).	Smoking, alcohol, poor hygiene and high jaw dose predict ORN; stresses long-term follow-up and stronger pre-RT protocols.
Zadik et al. 2025 (MASCC/ISOO CPS)	Mucositis risk; ORN	Complete dental clearance; remove infection foci; extractions with 2-3 wk healing; professional hygiene + CHX; home/office fluoride; antibiotics in severe neutropenia; G-CSF support; dosimetry-guided extractions for RT >44 Gy.	Reduces infections, OM exacerbation and treatment interruptions; worse outcomes when dental needs untreated pre-HCT.	Hematologic/CAR-T focus; practical algorithm rather than detailed mucositis-drug guideline.

Summary of therapeutic approaches across the 12 included protocols. See Supplementary Table S1 (http://www.medicina.oral.com/carpeta/suppl1_27886) for full details. OM: Oral mucositis; ORN: Osteoradionecrosis; RT: Radiotherapy; HNC: Head-and-neck cancer; HSCT/HCT: Hematopoietic stem-cell transplantation; CAR-T: Chimeric antigen receptor T cells; CHX: Chlorhexidine; NaF: Sodium fluoride; PBM: Photobiomodulation; HBO: Hyperbaric oxygen.

## Data Availability

Declared none
